# The relationship between pre-operative psoas and skeletal muscle parameters and survival following endovascular aneurysm repair: a systematic review and meta-analysis

**DOI:** 10.1038/s41598-022-20490-3

**Published:** 2022-10-05

**Authors:** N. A. Bradley, C. S. D. Roxburgh, D. C. McMillan, G. J. K. Guthrie

**Affiliations:** Academic Department of Surgery, University of Glasgow, New Lister Building, Glasgow Royal Infirmary, Glasgow, G4 0SF UK

**Keywords:** Medical imaging, Cardiovascular diseases, Aneurysm, Risk factors

## Abstract

Sarcopenia is characterised by chronically reduced skeletal muscle volume and function, and is determined radiologically by psoas and skeletal muscle measurement. The present systematic review and meta-analysis aims to examine the relationship between pre-operative CT-derived psoas and skeletal muscle parameters and outcomes in patients undergoing EVAR and F/B-EVAR for aortic aneurysm. The MEDLINE database was interrogated for studies investigating the effect of pre-operative CT-diagnosed sarcopenia on outcomes following EVAR and F/B-EVAR. The systematic review was carried out in accordance with PRISMA guidelines. The primary outcome was overall mortality. RevMan 5.4.1 was used to perform meta-analysis. PROSPERO Database Registration Number: CRD42021273085. Ten relevant studies were identified, one reporting skeletal muscle parameters, and the remaining nine reporting psoas muscle parameters, which were used for meta-analysis. There were a total of 2563 patients included (2062 EVAR, 501 F/B-EVAR), with mean follow-up ranging from 25 to 101 months. 836 patients (33%) were defined as radiologically sarcopenic. In all studies, the combined HR for all-cause mortality in sarcopenic versus non-sarcopenic patients was 2.61 (1.67–4.08), *p* < .001. Two studies reported outcomes on patients undergoing F/B-EVAR; the combined HR for all-cause mortality in sarcopenic versus non-sarcopenic patients was 3.08 (1.66–5.71), *p* = .004. Radiological sarcopenia defined by psoas or skeletal muscle parameters was associated with inferior survival in patients undergoing both EVAR and F/B-EVAR. Current evidence is limited by heterogeneity in assessment of body composition and lack of a consensus definition of radiological sarcopenia.

## Introduction

The estimated UK prevalence of abdominal aortic aneurysm (AAA) is 1.5%, rising to 1.9–4.0% in males aged over 65^1,2^. The 2019 National Vascular Registry Annual Report stated that 63% of UK aneurysms were managed by endovascular aneurysm repair (EVAR)^3^. Complex EVAR, either fenestrated EVAR or branched EVAR (F/B-EVAR), are increasingly the treatment choice for juxta- or para-renal aneurysms, or thoracoabdominal (TAAA) aneurysms. Complex endovascular repair is reported to offer superior perioperative mortality rates when compared with conventional open repair of complex aneuryms^4,5^. The safety profile of F/B-EVAR has resulted in an increase in older patients being offered complex endovascular repair^6,7^.

Sarcopenia is characterised by loss of skeletal muscle mass and function, and is associated with frailty, poor functional status, inferior physiological reserve, increasing age, and chronic illness^8,9^. There is a well-defined relationship between sarcopenia and inferior perioperative outcomes and in a variety of surgical specialties^10,11^. Sarcopenia has been reported to be prevalent in cardiovascular disease and particularly in the vascular surgical population^12^. Sarcopenia can be assessed through CT-derived body composition analysis^13^. This is typically performed by assessment of cross-sectional muscle area, leading to a diagnosis of “radiological sarcopenia”^14^. The assessment of psoas muscle area (PMA) or psoas muscle index (PMI; PMA normalised to height^2^), or skeletal muscle area/index (SMA/SMI) at the L3 vertebral level has been reported and validated as predictive of post-operative outcomes in a range of surgical conditions^13,15^. SMI offers superior prognostic value than PMI in general surgical cohorts^16^, however in vascular surgical populations psoas muscle has, to date, been predominantly reported. Skeletal muscle attenuation/density (SMAt/SMD) is inversely proportional to intramuscular fat deposition (myosteatosis) and has been reported as a marker of muscle quality^17^, however to date this parameter is unreported in relation to prognosis in patients with AAA.

A previous meta-analysis^18^ included seven observational studies with a total of 1440 patients undergoing OSR or infrarenal EVAR for AAA in whom preoperative measurements of psoas muscle had been performed^18^. They reported an overall increased risk of post-operative mortality associated with low PMA/PMI (HR 1.66, 95% CI 1.15–2.40; *p* = 0.007) in their entire (combined OSR and EVAR) study cohort. Subgroup analysis of the EVAR patients showed a trend towards increased mortality in patients with low PMA/PMI, though this was non-significant (HR 1.86, 95% CI 1.00–3.43; *p* = 0.05).

Subsequent observational studies have reported inferior survival outcomes in cohorts consisting of only patients undergoing EVAR, and in patients undergoing F/B-EVAR, though these results have not been reproducible. A lack of a standardised definition of radiological sarcopenia in patients with AAA has resulted in a heterogenous evidence base with variable definitions of sarcopenia. A recent meta-analysis^19^ described a prognostic role for pre-operative sarcopenia in relation to 5-year mortality, however only included three studies (604 patients) in quantitative meta-analysis^19^. Furthermore, Dakis et al. did not included patients undergoing F/B-EVAR in their analysis; this patient group is typically less frail than patients undergoing standard EVAR and the incidence and prognostic value of sarcopenia in this setting is uncertain.

The present systematic review aims to examine the relationship between pre-operative CT-derived psoas and skeletal muscle parameters and clinical outcomes in patients undergoing EVAR and F/B-EVAR for AAA.

## Materials and methods

The present review and search strategy was carried out in accordance with Preferred Reporting Items for Systematic Reviews and Meta-Analyses (PRISMA) guidelines. The review protocol was registered with the PROSPERO database (Registration Number: CRD42021273085), and the review protocol can be accessed via the PROSPERO database. No patient data were accessed, therefore specific ethical approval was not required.

Studies meeting the following eligibility criteria were included:

### Inclusion criteria

The following criteria were applied to determine eligibility: retrospective and prospective study design, study population including patients undergoing standard EVAR or complex (F/B-EVAR) endovascular aneurysm repair in the elective and emergency setting, peri-operative CT-imaging used to perform muscle group analysis, studies using CT imaging to calculate PMA/PMI/SMA/SMI with an accepted technique (as reported in previous literature), all-cause mortality reported in relation to PMA/PMI/SMA/SMI using a pre-determined threshold to subgroup patients into “sarcopenic” and “non-sarcopenic” with comparison of survival outcomes (either time to event analyses and log-rank t tests or HR and 95% CI) between the two cohorts, all follow-up duration.

### Exclusion criteria

The following criteria were applied to determine ineligibility: study population including patients undergoing EVAR or F/B-EVAR for non-aneurysmal disease of the aorta, or isolated iliac artery aneurysms, studies including paediatric patients (age < 18).

### Outcomes

Meta-analysis was performed on primary and secondary outcomes; primary outcome was defined as overall post-operative survival during the follow-up period, and secondary outcome was early (inpatient or < 30 day) mortality. Qualitative analysis of included studies was also performed as part of the present review.

### Search strategy

The MEDLINE database was accessed electronically using the PubMed (National Center for Biotechnology Information, U.S. National Library of Medicine, Bethesda MD, USA) search engine. The search was conducted on the 12th January 2022; any papers published after this date are not included in the present review.

The operator “AND” was used to combine search terms. Four search terms were used; “sarcopenia” AND “EVAR”, “psoas” AND “EVAR”, “sarcopenia” AND “endovascular”, “psoas” AND “endovascular”. Each of these search terms were applied to study title, key words, and Medical Subject Heading (MeSH) terms. Duplicate results were screened by identifying their PubMed Identifier (PMID), an integer value unique to each record.

The search was reproduced by two independent investigators (NB/GG). Each investigator screened study abstracts and excluded studies which did not meet the previously determined inclusion criteria. Full papers were subsequently screened and again exclusions made. A list of eligible studies was generated by each investigator, and both lists were compared. Discrepancies between the two lists were resolved by discussion and agreement.

Data were extracted from full text articles, tables, figures, and (where applicable) appendices. Data were recorded using Microsoft Excel.

### Data extracted from studies

The following data were extracted from each study: study design (centres, follow-up, prospective/retrospective) and study information (journal, authors, year), baseline clinical (comorbidities, routine pre-operative blood results) and demographic data of patients, data relating to procedure-specific factors (*n* standard/complex EVAR and emergency/elective), technique of measuring muscle area from pre-operative CT, method used to define sarcopenic patients and *n *of sarcopenic and non-sarcopenic patients (e.g. based on previous literature, data-dependent ROC analysis/tertiles), survival data for overall survival during study & early mortality, length of hospital stay.

### Risk of bias assessment

Risk of bias in the selected studies was assessed by applying the stepwise criteria in the Quality in Prognostic Studies (QUIPS) tool (Appendix 1)^20^. Domains assessed by this tool are Study Participation, Study Attrition, Prognostic Factor Measurement, Outcome Measurement, Study Confounding, and Statistical Analysis and Reporting, with each domain graded as High, Moderate, or Low.

### Data extraction

Data extraction was direct where possible; for the primary outcome (overall survival during study follow-up period) this was extraction of Hazard Ratio (HR), *p* value, and 95% CI generated by Cox Proportional Hazards Model comparing sarcopenic vs. non-sarcopenic patients. Where the covariate of interest was reported in both univariate and multivariate analyses, the HR generated by multivariate analysis was chosen. Where studies did not report HR and 95% CI indirect data extraction was performed; time to event Kaplan–Meier plots were digitised using WebPlotDigitizer program^21^ and survival estimates calculated. HR and 95% CI were calculated from the study-reported numbers at risk and calculated survival estimates using the method described by Tierney et al.^22^. Both directly and indirectly extracted HR and 95% CI were subsequently used to calculate ln[HR] and standard error, from which a Forest Plot with each study's effect size was formed using the generic inverse variance method.

For the secondary outcome early mortality % of events and non-events (deaths < 30 days post-op) were directly extracted from studies and a dichotomous model used to calculate OR for each study. Where authors reported length of follow-up in years, this was converted to months by the study team.

### Meta-analysis

The effects observed from meta-analysis will be generalised outside of the included studies. Based on preliminary literature review, heterogeneity between studies was expected (variation in the number of each type of repair, or different definitions of low PMI between studies) therefore a random effects model was used for meta-analysis. Heterogeneity was quantitatively assessed using I^2^ statistic, with values greater than 25%, 50%, and 75% representing low, moderate, and high heterogeneity as per Higgins et al.^23^. Review Manager (RevMan) Version 5.4.1 (Cochrane Collaboration)^24^ was used to compile and analyse data for meta-analysis.

### Subgroup analyses

The subgroup “complex EVAR” (patients undergoing F/B-EVAR) were separately analysed to assess the effect of low PMI on survival in this cohort. It was anticipated that each study included should clearly state the numbers of patients undergoing this type of procedure as the risk profile and clinical course are heterogenous compared to standard EVAR.

## Results

### Study selection

**Figure 1:**
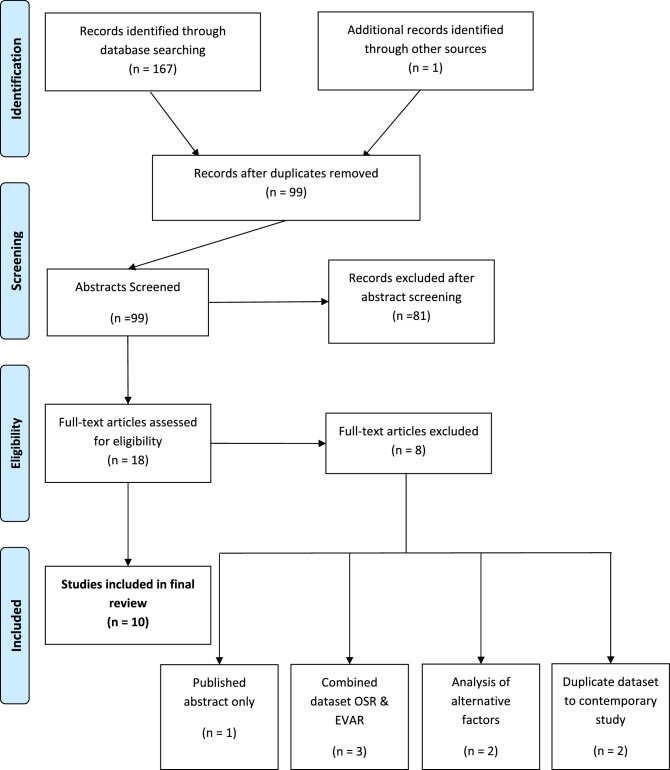
PRISMA diagram showing study inclusion. OSR (Open Surgical Repair), EVAR (Endovascular Aneurysm Repair), SMI (Skeletal Muscle Index), PMI (Psoas Muscle Index).

The study selection process is summarised in the PRISMA diagram in Fig. 1. The search terms “sarcopenia” AND “EVAR”, “psoas” AND “EVAR”, “sarcopenia” AND “endovascular”, “psoas” AND “endovascular” returned 14, 28, 39, and 85 records respectively. One additional record was obtained through review of citations from a previous meta-analysis^18^. This gave an initial total of 167 records.

When duplicate records were removed, 99 records remained, which underwent abstract screening. 81 records were excluded based on abstract screening based on non-eligibility as per inclusion and exclusion criteria. 18 full papers were reviewed for eligibility and eight were subsequently excluded; three performed analysis only on a combined dataset of patients undergoing OSR and EVAR, two reported analysis of factors (change in PMI) other than baseline PMA/PMI/SMA/SMI, two were studies performed on institutional datasets which had subsequently or previously been reported elsewhere which better fit the eligibility criteria (removed to eliminate duplicated patients being included), and one was a published abstract only. This left 10 studies included in the final review process, nine of which were observational retrospective studies, and one of which (Waduud et al.) was an observational prospective study^25–34^.

Of the 10 included studies, to define radiological sarcopenia, nine used measures of psoas muscle (PMA/PMI) and one used measures of total skeletal muscle (SMA). Due to heterogeneity in these methods of assessment, the study measuring SMA (Hale et al.^34^) was not included in meta-analysis; qualitative review was instead performed.

### Study characteristics

A summary of the included studies is shown in Table 1. There were a total of 2563 patients included (2062 EVAR, 501 F/B-EVAR), of which 836 (33%) were defined as having radiological sarcopenia (585 EVAR (28% of total), 251 F/B-EVAR (50% of total)).One study (Oliveira et al.^26^) excluded female patients, whilst the remaining studies included both males and females. Inclusion and exclusion criteria were otherwise similar in all studies; all authors excluded patients undergoing procedures for non-aneurysmal disease. There were no emergency cases included. The mean follow-up period ranged from 25.2 to 100.8 months in 8 studies, and was not specifically reported in two studies. Eight studies were single centre, one was multicentre, and in one the dataset was based on interrogation of national registry data.

**Table 1:** Studies included in the final review of the prognostic effect of radiological sarcopenia in patients undergoing EVAR and F/B-EVAR.

Study	*n*	Definition of “Sarcopenic” cohort	*n* sarcopenic	Normalisation?	Follow-up (mean)	Data extraction	Centres	Technique of muscle analysis (Software, Thresholds, Level)	Timing of CT
Ikeda et al.^25^	324 elective EVAR	PMA, ROC analysis	166	No	56.7 months	Direct (HR)	Single	Aquarius (manual) NR L4	NR
Oliveira et al.^26^	105 elective EVAR**	PMA; Lowest tertile	35	No	27.6 months	Direct (HR)	Single	Osirix (semi-automated) − 29 to + 150 L3	NR
Thurston et al.^27^	191 elective EVAR	PMI < 500 mm^2^/m^2^	30	Height^2^	NR	Direct (HR)	Multi	Osirix (semi-automated) NR L3	NR
Huber et al.^28^	407 elective EVAR	PMA; Lowest quartile	102	No	39.0 months	Direct (HR)	Single	Carestream (manual) NR L4	NR
Kärkkäinen et al.^29^	244 elective F/B-EVAR	PMI; adjusted hazard model defined threshold	165	Height^2^	25.2 months	Indirect	Single	QReads (manual) NR L3	NR
Ito et al.^30^	310 elective EVAR	PMI; 30th Percentile, ROC defined threshold	93	Height^2^	35.3 months	Direct (HR)	Single	Synapse Vincent (semi-automated) NR L3	< 1 month pre-operatively
Alenezi et al.^31^	257 elective F/B-EVAR	PMA; Lowest tertile	86	No	32.7 months	Direct (HR)	Single	Coral Ris (manual) NR L3	< 12 months pre-operatively—< 1 week post-operatively
Cheng et al^32^	272 elective EVAR	PMI; ROC defined threshold	50	Height^2^	NR	Indirect	Single	Centricity (semi-automated) NR L3	< 90 days pre-operatively—< 30 days post-operatively
Waduud et al.^33^	253 elective EVAR	PMA; Lowest tertile	84	No	48.0 months	Direct (HR)	Registry Data	Impax (manual) NR L3	< 12 months pre-operatively
Hale et al^34^	200 elective EVAR	SMA; < 114.0 cm^2^ (men)/< 89.8 cm^2^ (women)	25	No	100.8 months	N/A	Single	AGFA PACS (manual) NR L3	“just prior” to procedure—< 1 month post-operatively

*NR* not reported, *HR* hazard ratio.

**Excluded female patients.

### Risk of bias in included studies

The risk of bias assessment using the QUIPS tool is shown in Table 2. Full risk of bias assessment proforma for each of the included studies is shown in Appendix 1.

**Table 2:** Risk of bias summary judgements (from QUIPS tool) for the studies included in the final review.

Study	Study participation	Study attrition	Prognostic factor measurement	Outcome measurement	Study confounding	Statistical analysis and reporting
Ikeda et al. ^25^	Moderate	Low	Moderate	Moderate	Moderate	Low
Oliveira et al.^26^	High	High	Moderate	Moderate	Moderate	Low
Thurston et al.^27^	Moderate	Low	Low	Moderate	Moderate	Low
Huber et al.^28^	Moderate	Low	Moderate	Moderate	Moderate	Moderate
Kärkkäinen et al.^29^	Moderate	Low	Moderate	Low	Moderate	Low
Ito et al.^30^	Low	Low	Moderate	High	Moderate	Low
Alenezi et al.^31^	Moderate	Low	High	Moderate	Moderate	Low
Cheng et al^32^	Moderate	Low	Moderate	Low	Moderate	Low
Waduud et al.^33^	Low	Moderate	Moderate	Low	Moderate	Low
Hale et al.^34^	Moderate	High	Low	Low	Moderate	Low

### Meta-analysis of primary outcome

**Figure 2:**
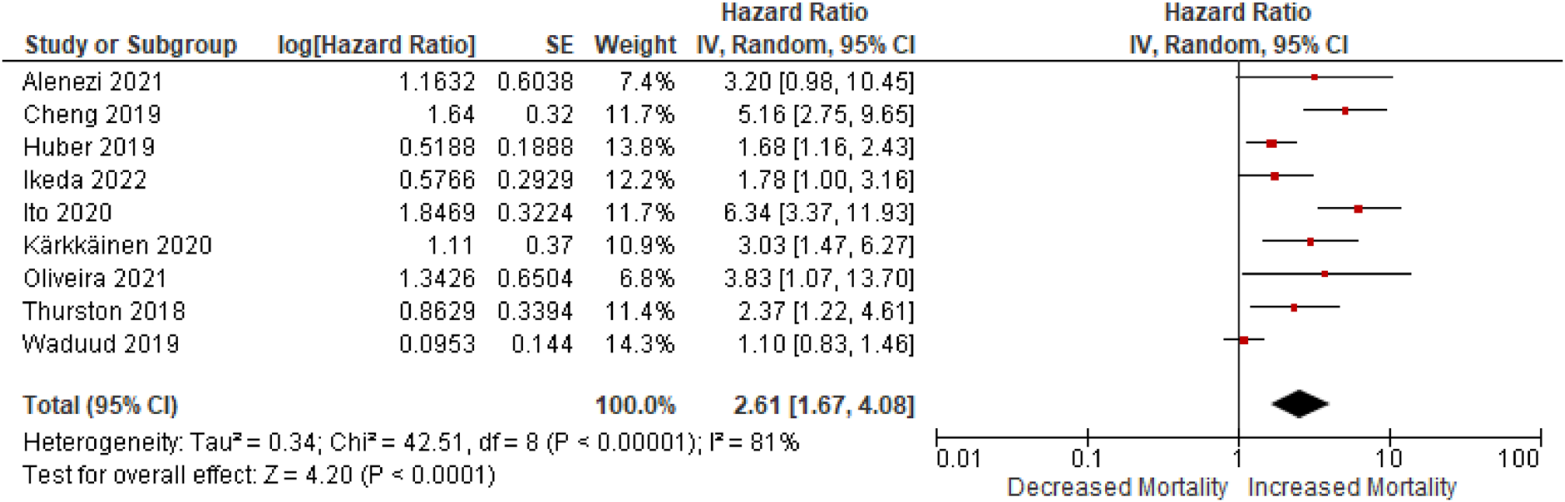
Forest Plot of the risk of post-operative mortality in sarcopenic vs. non-sarcopenic patients undergoing EVAR and F/B-EVAR for each of the included studies. Plots to the right of the X-axis represent an increased risk of mortality in sarcopenic patients.

Nine studies reported psoas muscle parameters in 2363 patients which were extracted for meta-analysis of primary outcome; seven of these were direct extraction of hazard ratios in sarcopenic vs. non-sarcopenic patients, and two were indirect extraction from time-to-event curves. A Forest Plot for overall survival is shown in Fig. 2. The combined HR for all-cause mortality in sarcopenic versus non-sarcopenic patients was 2.61 (1.67–4.08), *p* < 0.00001. Heterogeneity between studies was high (I^2^ = 81%).

### Meta-analysis of secondary outcome

**Figure 3:**
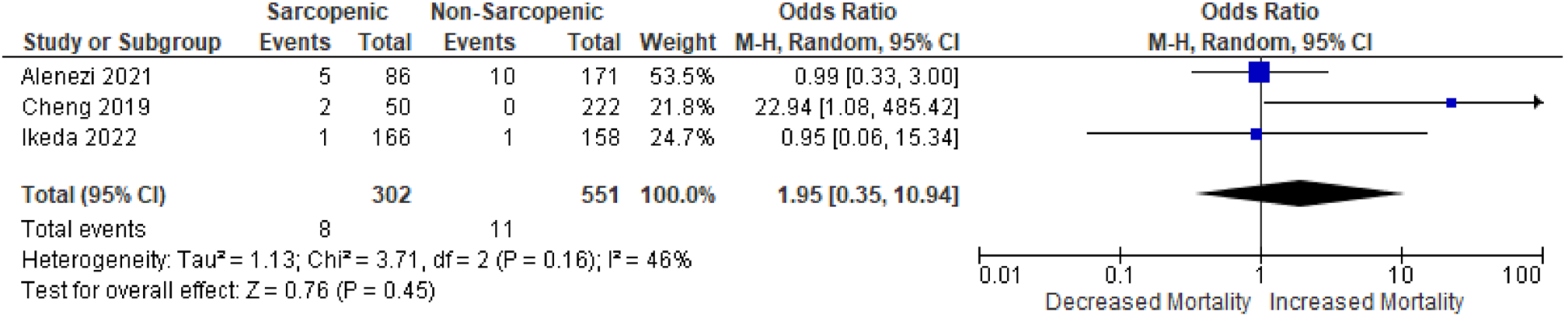
Forest Plot of the risk of 30 day post-operative mortality in sarcopenic vs. non-sarcopenic patients undergoing EVAR and F/B-EVAR in the included studies. Plots to the right of the X-axis represent an increased risk of mortality in sarcopenic patients.

Three studies reported early mortality in 853 patients and were included in meta-analysis. Of the remaining studies, two did not report early mortality, two reported overall cohort early mortality without specifying the difference based on muscle area analysis, and one did not report any early deaths. A Forest Plot for early mortality is shown in Fig. 3. The combined HR for early mortality in sarcopenic versus non-sarcopenic patients was 1.95 (0.35–10.94), *p* = 0.45. Heterogeneity between studies was low (I^2^ = 46%).

### Meta-analysis of subgroups

**Figure 4:**
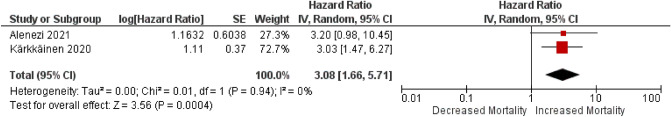
Forest Plot of the risk of post-operative mortality in sarcopenic vs. non-sarcopenic patients undergoing F/B-EVAR for the included studies. Plots to the right of the X-axis represent an increased risk of mortality in sarcopenic patients.

There were two studies which reported primary outcome in F/B-EVAR patients (Table 1). A Forest Plot for overall survival is shown in Fig. 4. The combined HR for all-cause mortality in sarcopenic versus non-sarcopenic patients was 3.08 (1.66–5.71), *p* = 0.004. Heterogeneity between studies was low (I^2^ = 0%).

### Skeletal muscle area measurement

One study reported SMA as the variable of interest; Hale et al. retrospectively analysed a prospectively recruited cohort of 200 patients undergoing elective standard EVAR^34^. Analysis was defined on absolute values of SMA; SMA < 114.0 cm^2^ (men)/ < 89.8 cm^2^ (women), based on values derived from patients undergoing liver transplantation and subsequently validated in patients with critical limb ischaemia^35,36^. Median follow-up was 100.8 months. Patients in the low SMA cohort (n = 25, 13%) were older and more likely to be female, with other baseline variables similar between the group. Overall mortality during the follow-up period was higher in the low SMA cohort (76% vs. 48%, *p* = 0.016). Low SMA status was associated with increased odds of mortality on multivariate analysis (OR 3.17, 95% CI 1.20–9.54), however this was not independent of age at the time of procedure.

### Timing of CT (Table 1)

Timing of CT from which images were extracted for subsequent analysis was heterogenous across the included studies. Five studies did not specify timing of CT in relation to the procedure. Two studies included only pre-operative CTs, from < 12 and < 1 months prior to the procedure. Three studies included both pre- and post-operative CTs, ranging from < 12 months pre-operatively to < 1 month post-operatively.

### Software used for analysis (Table 1)

There was heterogeneity in the choice of software used to analyse muscle areas. Four studies used semi-automated techniques (Osirix, Synapse Vincent, Centricity). The remaining six studies used manual techniques, with each of these studies using different software (Aquarius, Carestream, QReads, Coral Ris, Impax, AGFA PACS).

### Thresholds for skeletal muscle (Table 1)

Of the 10 included studies only one (Oliveira et al.^26^) specified the thresholds of HU used to define muscle tissue for body composition analysis; the widely accepted thresholds − 29 to + 150 HU were used by these authors.

### Normalisation (Table 1)

Five of the 10 included studies normalised PMA measurements to patient height^2^ to produce PMI, five studies reported data without normalisation in the form of PMA (4 studies) and SMA (one study).

### Thresholds used to stratify patients (Table 1)

Two studies used absolute values of muscle area to stratify patients for survival analysis; Thurston et al. used PMI < 500 mm^2^/m^2^, and Hale et al. used SMA < 114.0 cm^2^ (male patients)/ < 89.8 cm^2^ (female patients)^27,34^. Eight studies used thresholds derived from their own population data; tertiles/quartiles were used in four studies, whilst four used ROC analysis to determine optimal threshold based on sensitivity.

## Discussion

The present systematic review and meta-analysis shows that, in patients undergoing elective standard and complex EVAR to treat AAA, radiological sarcopenia, as defined by low PMA/PMI/SMA, was associated with significantly poorer survival outcome with a trend towards increased risk of early mortality. However, the number of studies/patients identified was small, there was considerable heterogeneity in the meta-analysis, poorly described methodology and study quality with considerable risk of bias. For example, approximately half of the studies identified did not describe their methodology in detail and/ or normalise their results to patient height^2^, which compromised comparison between studies. Therefore, further studies of the prognostic value of skeletal mass and quality in patients undergoing EVAR to treat AAA are warranted.

This relationship between CT-derived low PMI and outcomes in patients with AAA undergoing OSR has previously been described by Antoniou et al. in a 2019 meta-analysis^18^. Subgroup analysis performed by these authors demonstrated a trend towards inferior survival in the EVAR-only cohort. Dakis et al. recently reported a similar review of studies to the present study^19^. However, they excluded 73% of the studies in their review from meta-analysis, due to significant heterogeneity in outcome measures included studies. We note that despite their study selection, heterogeneity in meta-analysis remained high (I^2^ = 95.05%). Dakis et al. performed meta-analysis on 5 year survival, resulting in the exclusion of the majority of studies. Given that patients undergoing endovascular repair may have been selected to not undergo open surgical repair due to fitness and comorbidity, reporting outcomes at less than 5-year follow-up is clinically relevant and therefore the results observed in the present study support and complement those observed by Dakis et al.

The studies included in the previous and the present meta-analyses were limited by the lack of a consensus definition of body composition parameters to define radiological sarcopenia in patients with AAA. Moreover, there was significant heterogeneity in the muscle group analysed, lack of normalisation of muscle areas, limited information on the timing of CT from which measurements are taken, thresholds used to determine the “sarcopenic” cohort and software used to perform muscle area analysis. The greatest disparity in hazard ratios was observed between two groups of studies; Huber et al. and Waduud et al. vs. Cheng et al. and Ito et al.^28,30,32^^,^^33^. The former studies use manual software to measure muscle areas, report non-normalised (to height) values of muscle parameters, and use percentile-based (quartile, tertile) thresholds to define sarcopenia. The latter studies use semi-automated techniques, report normalised values of psoas muscle parameters, and use thresholds based on sensitivity and specificity (ROC) of their own data. The use of ROC analysis to define the sarcopenic cohort may introduce bias through maximising chance of observing a difference, which may account for the higher hazard ratios observed. In patients with cancer there are several proposed thresholds for different body composition parameters^14,37–39^, which have been subsequently applied to different patient groups. To date these thresholds have not been applied to patients with AAA, and their validity in this patient group is lacking. Further work to either derive novel thresholds or apply existing thresholds to patients with AAA is required.

Variation in body composition analysis between different software packages has been described, with several authors reporting that manual segmentation yields higher values of cross sectional area^40^. The advantages and disadvantages of each software are based on a variety of factors, such as speed of measurement, cost, and learning curve required.

Furthermore, eight studies used thresholds derived from their own datasets to define sarcopenia and only one study^13^ used a threshold derived from another cohort. This may have limited the conclusions derived from the present meta-analysis. There are several widely used thresholds used in cancer patients derived from large cohort studies however to date such data are not available in patients with AAA^14,38,41^.

The choice of muscle group analysed was psoas muscle (PMA/PMI) in nine of the included studies, with only one study performing analysis of total skeletal muscle area (SMA). There may be significant variation in the utility of PMI or SMI as a prognostic marker. For example, SMI has been shown to better predict outcomes in non-AAA populations^16,42^. To date PMA/PMI has been more widely utilised in AAA populations, perhaps due to less anatomical complexity and the less time-consuming measurement process^43^. Radiological assessment of abdominal muscle groups may be limited in the case of ruptured AAA, with retro- or intra-peritoneal haematoma precluding accurate delineation of muscle borders. Therefore, variations in the above factors may have limited the inter-study comparison performed in the present review.

The present study included both patients undergoing EVAR and F/B-EVAR. Whilst there may be heterogeneity between technical procedural differences and aneurysm morphology, we would not expect there to be significant difference in the underlying pathophysiology of the aneurysmal disease. Indeed, risk factors between patients with infra- and juxta-/para- renal AAA are likely to be similar. Whilst periprocedural complications are known to be different between the 2 interventions^7^, these may not extend to alterations in long-term mortality as observed by the present review. We hypothesise that the hazard of mortality in patients with AAA is reflective of the underlying cardiovascular morbidity in this patient group. Whilst AAA is not a primary atherosclerotic pathology, the risk factors for atherosclerotic disease are common to those for AAA, and a large proportion of patients with AAA may have systemic manifestations of atherosclerosis^44^. Sarcopenia in this setting may be reflective of more severe underlying generalised cardiovascular disease and this may explain the prognostic implications. Whilst patients undergoing F/B-EVAR typically have more complex aneurysms, it remains unclear whether aneurysmal extent itself is a prognostic factor in long-term mortality.

The contemporary definition of sarcopenia (EWGSOP2) includes not only loss in skeletal muscle volume but also reduced skeletal muscle function^9^. This has been reported in patients with cancer as the CT-derived SMD^17^. The prognostic value of SMD in patients with AAA is unreported, and requires further investigation in order to determine its clinical utility.

NICE guidance recommends offering open surgical repair (OSR) unless there is a contra-indication: including co-pathology; anaesthetic risk; and/or medical pathology^45^. Those patients who do not meet the fitness criteria to undergo OSR may be offered an endovascular procedure if aneurysm morphology is suitable. Therefore, it may be anticipated that the prevalence of low PMI would be higher in patients undergoing EVAR compared with those undergoing OSR. However, Waduud et al. report a higher PMA in patients undergoing EVAR, whilst Drudi et al. report a higher proportion of patients in their low PMI tertile undergoing EVAR^33,46^.

Subgroup analysis in the present study demonstrated inferior survival in sarcopenic patients undergoing F/B-EVAR, in keeping with the findings in standard EVAR. The increased risk of morbidity following F/B-EVAR compared to EVAR is well established^7,47^. Only 501 (19.5%) of patients in the present study underwent F/B-EVAR, with meta-analysis weighted heavily (72.7%) towards a single study. The lack of reporting on such patients limits the ability to draw meaningful conclusions from meta-analysis, however we believe it concisely highlights the paucity of evidence.

The results of the present study did not support increased early mortality in patients with sarcopenia. The maximal physiological compromise following surgery is expected to occur in the immediate post-operative period, where the level of inflammatory cytokines responding to the surgical insult peak and then gradually decline^48,49^. Stent-graft implantation provokes an inflammatory reaction, with variable cytokine response depending on graft material used^50^. This is manifest as the clinical “Post-Implantation Syndrome”, which has been reported to be associated with increased early mortality, though there is a paucity of long-term follow-up data. There is a well-described pro-inflammatory component of sarcopenia^51^, as is the relationship between chronic inflammation and increased cardiovascular mortality and morbidity^52,53^. There appears to be a complex relationship between chronic inflammation in patients with sarcopenia though this remains poorly defined in patients with AAA. Further characterisation of the inflammatory pathways in this patient group, including the effect of stent-graft deployment, is required.

The implication of these findings on the decision to proceed to repair in patients with sarcopenia may yield clinical benefit. Rate of aneurysm rupture in AAA 5.5–6.0 cm which are untreated has recently been reported as 2.2–3.5%, lower than previously thought^54,55^. Whilst the results of the present study are insufficient to directly apply body composition analysis to the clinical setting, we demonstrate that the derivation and validation of thresholds for muscle area to define patients as sarcopenic is required, as well as standardisation of measurement and reporting techniques. Both NICE^45^ and ESVS^56^ highlight the challenges in pre-operative assessment in their most recent guidelines which emphasise selective use of pre-operative testing at the discretion of clinicians. The use of body composition may complement existing methods of pre-operative risk assessment in this patient group.

## Supplementary Information

Supplementary Information.

## Data Availability

Data relating to the production of this manuscript are available on and can be obtained by contacting the corresponding author (NAB).
